# Nasopharyngeal carcinoma incidence and mortality in China, 2013

**DOI:** 10.1186/s40880-017-0257-9

**Published:** 2017-11-09

**Authors:** Kuang-Rong Wei, Rong-Shou Zheng, Si-Wei Zhang, Zhi-Heng Liang, Zhu-Ming Li, Wan-Qing Chen

**Affiliations:** 1 Cancer Institute of Zhongshan People’s Hospital, Zhongshan, Guangdong 528403 P. R. China; 2 National Central Cancer Registry, National Cancer Center, Beijing, 100021 P. R. China

**Keywords:** Nasopharyngeal carcinoma, Incidence, Mortality, China

## Abstract

**Background:**

We estimated the incidence and mortality of nasopharyngeal carcinoma (NPC) in China in 2010 according to the data of 145 domestic population-based cancer registries in 2014, and no such reports since then. Hence, to further and better understand its epidemiology in China and to provide more precise scientific information for its control and prevention in China, we analyzed the NPC incidence and mortality of 255 domestic population-based cancer registries, and estimated the national rates in 2013 again.

**Methods:**

NPC incidence and mortality data of 255 domestic cancer registries in 2013, accepted by the 2016 National Cancer Registry Annual Report, were collected and collated, and the indices of NPC such as the numbers of new cases and deaths, crude rates, age-standardized rates, and truncated rates of incidence and mortality were calculated and analyzed. The incidence and mortality in China and its constituent areas were estimated according to the national population in 2013.

**Results:**

An estimated 42,100 new cases and 21,320 deaths were attributed to NPC in China in 2013, accounting for 1.14% of all new cancer cases and 0.96% of all cancer-related deaths that year in China. Crude incidence and mortality of NPC were 3.09/100,000 and 1.57/100,000, respectively. World age-standardized incidence and mortality were 2.17/100,000 and 1.08/100,000, respectively. The incidence and mortality of males were obviously higher than those of females and slightly higher in urban areas than in rural areas. Among seven Chinese administrative regions, NPC incidence and mortality were obviously higher in South China than in other regions and lowest in North China. Top 3 incidence and mortality provinces and registering areas all located in South China. The age-specific incidence and mortality rose quickly from age 25–29 and 35 to 39 years, respectively, peaked at different ages and varied by location.

**Conclusions:**

These results demonstrated that NPC incidence and mortality in China in 2013 were also at high levels worldwide, which suggested that its control and prevention should be enhanced.

In 2014, for the first time in China, we estimated NPC incidence and mortality in whole China according to the data of 145 domestic population-based cancer registries with high data quality and the national population in 2010 [1], and we could know more about the epidemiology of NPC in China, except the data of GLOBOCAN [2], Cancer Incidence in Five Continents (CIF) [3], World Health Organization (WHO) [4], the national all-death-causes retrospective sampling survey [5], and the domestic cancer registries [1, 6]. At the same time, some researches revealed that, during the past decade, incidence of NPC is gradually declining worldwide, even in endemic regions, and mortality has fallen substantially [7–10], although its incidence increased in some countries [11] and tumor histology such as non-keratinizing tumors [12]. The mortality in China had been steadily declining and would continue to drop in the next few years [13], but some high-risk areas in China witnessed different trends, such as an increasing death trend in Guangxi autonomous region [14], declined incidence and mortality in urban Guangzhou [15], a stable incidence in Sihui [16] and Zhongshan [17 **]**, and a declining death trend in Zhongshan [18]. As of now, the incidence and mortality data of NPC in whole China were few. Hence, to further and better understand its epidemiological situation in China and to provide more precise scientific information for its control and prevention, we estimated the incidence and mortality of NPC in China in 2013 again according to the data of 255 domestic population-based cancer registries.

## Data and methods

### Data source

The NPC incidence and mortality data in this study came from 255 domestic population-based cancer registries, whose data quality met the criteria of National Central Cancer Registry (NCCR) [the percentage of cases with morphological verification (MV%) > 66%, the percentage of cases with death certification only (DCO%) < 15%, mortality to incidence [M/I] ratio between 0.6 and 0.8, and the percentage of the diagnosis of unknown basis (UB%) < 5%] and accepted by the 2016 National Cancer Registry Annual Report. The 255 registries, including 88 in urban areas and 167 in rural areas, were distributed in 31 provinces, autonomous regions, and municipalities directly under central government of China [19, 20]. NPC is coded as C11 in the International Statistical Classification of Diseases and Related Health Problems 10th Revision (ICD-10) [21].

Population data came from the Statistical and/or Public Security Bureaus of the above mentioned cancer registering areas, which covered a population of 226,494,490 persons, including 114,860,339 (50.71%) males and 111,634,151 (49.29%) females, or 111,595,772 (49.27%) persons in urban and 114,898,718 (50.73%) in rural areas. The covered population accounted for 16.65% of total Chinese population by the end of 2013.

### Statistical indices and methods

Statistical indices included the numbers of NPC incident cases and deaths, proportions, crude rates, age-standardized rates (ASRs) by Chinese (ASRC) and world (ASRW) standard population (ASIRC and ASMRC represents the ASRC of incidence and mortality, respectively, and ASIRW and ASMRW represents the ASRW of incidence and mortality, respectively), cumulative rates (aged 0–74 years), truncated age-standardized rates (TASR) (aged 35–64 years), and age-specific rates, stratified by areas, regions, and genders. The statistical methods recommended by the Guideline for Chinese Cancer Registration [22] were adopted. The standard population of China in 2000 and that of the World in 1985 (Segi) were used to calculate ASRs [19, 20].

The cancer registering areas included in the present study were classified as urban and rural areas or Eastern, Middle, and Western areas and were divided into seven administrative regions: South China, North China, Central China, East China, Southwest China, Northwest China, and Northeast China according to the criteria of the National Bureau of Statistics [19]. The statistical indices were calculated for the above areas and regions. By multiplying the age-specific incidence and mortality with the population numbers of corresponding age groups in 2013 in above each stratification, NPC incident case and death numbers in each stratification were obtained, and the estimated incident cases and deaths counts in whole China were obtained by pooling the data [19, 20].

All statistical analysis was conducted by using SAS software (SAS Institute Inc., Cary, USA).

## Results

### Data quality

Data quality varied by cancer registering area in this study. Data quality in urban areas was higher than that in rural areas, with the data quality in males close to that in females. The data quality was the lowest for the registries in the Western area, and similar for the registries in the Middle and Eastern areas. The MV%, DCO%, M/I ratio, and UB% of NPC data from the above mentioned 255 registries were 75.12, 1.18, 0.52, and 0.65%, respectively; they were 77.20, 1.09, 0.51, and 0.35%, respectively, for urban registries, and 72.78%, 1.29%, 0.54%, and 0.99%, respectively, for rural registries (Table 1).

**Table 1:** Quality of nasopharyngeal carcinoma (NPC) data in 2013 from 255 Chinese cancer registries

Areas	Sex	M/I ratio	MV%	DCO%	UB%
All	Both	0.52	75.12	1.18	0.65
	Male	0.54	75.02	1.25	0.69
	Female	0.48	75.36	1.02	0.53
Urban areas	Both	0.51	77.20	1.09	0.35
	Male	0.53	77.36	1.26	0.37
	Female	0.45	76.78	0.66	0.28
Rural areas	Both	0.54	72.78	1.29	0.99
	Male	0.55	72.31	1.24	1.07
	Female	0.50	73.88	1.40	0.80
Eastern area	Both	0.46	75.62	1.31	1.76
	Male	0.47	75.98	1.39	1.83
	Female	0.45	74.82	1.12	1.57
Middle area	Both	0.46	75.74	2.15	0.22
	Male	0.47	75.73	2.01	0.16
	Female	0.42	75.85	2.20	0.34
Western area	Both	0.52	63.62	3.33	1.02
	Male	0.56	63.56	3.61	0.70
	Female	0.45	63.74	2.72	1.59

*M/I* mortality to incidence, *MV%* the percentage of cases with morphologically verification, *DCO%* the percentage of cases with death certification only, *UB%* the percentage of diagnosis of unknown basis

### Incidence estimation

About 42,100 new NPC cases, accounting for 1.14% of all new cancer cases in China in 2013, were estimated to occur in China that year; its crude incidence, ASIRC, and ASIRW were 3.09/100,000, 2.31/100,000, and 2.17/100,000, respectively. About 30,000 new male NPC cases, accounting for 1.47% of all new male cancer cases in China in 2013, were estimated to occur in China that year; its crude incidence, ASIRC, and ASIRW were 4.31/100,000, 3.26/100,000, and 3.07/100,000, respectively. About 12,000 new female NPC cases, accounting for 0.74% of all new female cancer cases in China in 2013, were estimated to occur in China that year; its crude incidence, ASIRC, and ASIRW were 1.81/100,000, 1.35/100,000, and 1.25/100,000, respectively. The male incidence was higher than the female incidence. The ASIRW of males in urban, rural, and all areas were 2.59, 2.29, and 2.46 times higher than those of females, respectively. The incidence in the Western area was the highest, followed by those in the Middle and Eastern areas; the incidence in the Middle area was slightly higher than that in the Eastern area (Table 2).

**Table 2:** NPC incidence in China in 2013

Areas	Sex	CR (1/10^5^)	Prop (%)	ASIRC (1/10^5^)	ASIRW (1/10^5^)	Cum rate (%)	TASR (1/10^5^)	Rank
All	Both	3.09	1.14	2.31	2.17	0.24	4.88	19
	Male	4.31	1.47	3.26	3.07	0.34	7.01	14
	Female	1.81	0.74	1.35	1.25	0.13	2.68	19
Urban areas	Both	3.33	1.17	2.40	2.23	0.24	5.09	20
	Male	4.73	1.58	3.43	3.21	0.35	7.37	14
	Female	1.87	0.70	1.35	1.24	0.13	2.71	19
Rural areas	Both	2.82	1.11	2.19	2.07	0.23	4.59	19
	Male	3.82	1.33	3.04	2.89	0.32	6.50	12
	Female	1.75	0.79	1.34	1.26	0.14	2.65	20
Eastern area	Both	2.93	0.96	2.07	1.93	0.21	5.52	19
	Male	4.14	1.23	2.94	2.76	0.31	8.17	14
	Female	1.71	0.61	1.21	1.11	0.12	2.67	20
Middle area	Both	2.89	1.15	2.25	2.15	0.24	4.77	21
	Male	3.98	1.42	3.16	3.02	0.34	6.47	15
	Female	1.74	0.78	1.33	1.26	0.14	2.95	20
Western area	Both	4.57	1.86	3.58	3.37	0.37	6.58	18
	Male	6.35	2.25	5.03	4.73	0.52	8.78	14
	Female	2.72	1.32	2.12	1.99	0.21	4.31	19

*CR* crude rate, *Prop* proportion, *ASIRC* age-standardized incidence rate by 2000 Chinese standard population, *ASIRW* age-standardized incidence rate by 1985 Segi’s world standard population, *Cum rate* cumulative rate for patients aged 0–74 years, *TASR* truncated age-standardized rate for patients aged 35–64 years, *Rank* rank of NPC incidence in all cancers’ incidences

### Age-specific incidence

**Fig. 1:**
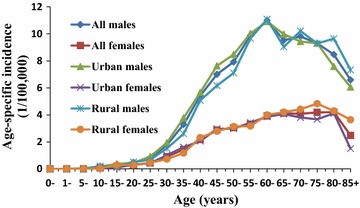
Nasopharyngeal carcinoma (NPC) age-specific incidence in China in 2013. The age-specific incidence increases remarkably from age 25–29 years, peaks at age 60–64 for males and age 75–79 for females, and decreases obviously thereafter. The incidences in males are much higher than those in females, but no obvious difference exists in age-specific incidence in either males or females between urban and rural areas

NPC age-specific incidences in China in 2013 began to increase quickly from age 25–29 years, peaked at age 60–64 for males and 75–79 years for females, and decreased obviously thereafter. The age-specific incidence in males was much higher than that in females. There was no obvious difference in the trends of male and female age-specific incidences between urban and rural areas (Fig. 1). Age-specific incidence varied by area at some degree, e.g., it peaked at age 80–84 years for males in the Western area and at age 85+ years for females in the Eastern area.

### Incidence differences between regions

An estimated 24,300 new NPC cases, accounting for 1.17% of all new cancer cases in Chinese urban areas in 2013, occurred in Chinese urban areas that year; the crude incidence, ASIRC, and ASIRW were 3.33/100,000, 2.40/100,000, and 2.23/100,000, respectively. An estimated 17,700 new NPC cases, accounting for 1.10% of all new cancer cases in Chinese rural areas in 2013, occurred in Chinese rural areas that year; the crude incidence, ASIRC, and ASIRW were 2.82/100,000, 2.19/100,000, and 2.07/100,000, respectively (Table 2). The incidences in urban areas were slightly higher than those in rural areas. The crude incidence, ASIRC, and ASIRW in urban areas were only 1.18, 1.10, and 1.03 times higher than those in rural areas, respectively.

Generally, the highest incidence was seen in the Western area, followed by the Middle and Eastern areas in China in 2013; the same trend was found in rural areas. In urban areas, the highest rate was seen in Western areas, followed by Eastern and Middle areas. However, no significant difference existed for the incidence among the above three areas.

Among the Chinese seven administrative regions, the incidence in South China was the highest, followed by those in Southwest, Central, East, Northwest, Northeast, and North China. Notably, the ASIRW in South China (9.69/100,000) was 3.4 times higher than that in Southwest China (2.85/100,000), in which the incidence was the second highest.

In terms of ASIRW, top 3 incidence provinces among 31 Chinese provinces, autonomous regions, and municipalities directly under the central government in 2013 were Guangxi, Guangdong, and Hunan, whose ASIRWs were 11.16/100,000, 10.38/100,000, and 5.38/100,000, respectively, all located in South China. Tibet, Sichuan, Chongqing, and Hubei also ranked top 10 incidence provinces, which do not locate in South China (Table 3). Top 3 incidence areas in the 255 domestic cancer registries in 2013 were Cangwu county of Guangxi autonomous region, Sihui city of Guangdong province, and Longnan county of Jiangxi province, whose ASIRWs were 25.39/100,000, 18.74/100,000, and 15.52/100,000, respectively. Top 10 incidence areas all located in South China (Table 4).

**Table 3:** Top 10 NPC incidence and mortality provinces, autonomous regions, and municipalities in China, 2013

Rank	Incidence (1/10^5^)				Mortality (1/10^5^)			
	Province^a^	CR	ASIRC	ASIRW	Province^a^	CR	ASMRC	ASMRW
1	Guangxi	12.37	11.16	10.49	Guangxi	5.83	5.16	5.01
2	Guangdong	12.30	10.38	9.62	Guangdong	6.26	5.06	4.99
3	Hunan	6.97	5.38	5.08	Hainan	4.44	3.27	3.27
4	Jiangxi	5.62	5.08	4.88	Hunan	3.76	2.88	2.71
5	Hainan	5.45	3.90	3.83	Jiangxi	2.89	2.53	2.44
6	Fujian	4.69	3.87	3.65	Guizhou	3.04	2.40	2.38
7	Tibet	2.66	3.15	3.06	Fujian	2.58	2.08	2.05
8	Sichuan	4.35	3.21	3.05	Sichuan	2.15	1.52	1.49
9	Chongqing	4.41	3.12	2.92	Shanghai	3.13	1.38	1.37
10	Hubei	4.46	3.04	2.88	Hubei	1.97	1.25	1.25

^a^Including provinces, autonomous regions, and municipalities directly under central government of China

*CR* crude rate, *ASIRC* age-standardized incidence rate by 2000 Chinese standard population, *ASIRW* age-standardized incidence rate by 1985 Segi’s world standard population, *ASMRC* age-standardized mortality rate by 2000 Chinese standard population, *ASMRW* age-standardized mortality rate by 1985 Segi’s world standard population

**Table 4:** Top 10 NPC incidence and mortality areas in China, 2013 (1/10^5^)

Rank	Incidence				Mortality			
	Area	CR	ASIRC	ASIRW	Area	CR	ASMRC	ASMRW
1	Cangwu county of Guangxi	27.13	26.95	25.39	Sihui city of Guangdong	20.19	14.28	14.24
2	Sihui city of Guangdong	25.26	20.91	18.74	Mayang county of Hunan	14.09	13.06	12.54
3	Longnan county of Jiangxi	14.42	16.04	15.52	Cangwu county of Guangxi	12.2	11.22	11.16
4	Zhongshan city of Guangdong	17.54	14.31	13.06	Zhongshan city of Guangdong	9.82	8.48	8.54
5	Nanxiong city of Guangdong	13.81	11.79	11.22	Longnan county	9.34	7.79	7.56
6	Jiangmen urban area of Guangdong	14.99	11.54	10.64	Fushui county of Guangxi	2.44	6.98	6.42
7	Mayang county of Hunan	14.77	10.94	10.53	Jiangmen urban area of Guangdong	9.31	6.34	5.96
8	Beiliu city of Guangxi	11.04	11.24	10.27	Hepu county of Guangxi	5.7	5.81	5.55
9	Fushui county of Guangxi	11.58	10.92	10.23	Beiliu city of Guangxi	3.87	4.50	4.50
10	Hepu county of Guangxi	11.74	10.34	9.96	Nanxiong city of Guangdong	9.57	4.32	4.31

*CR* crude rate, *ASIRC* age-standardized incidence rate by 2000 Chinese standard population, *ASIRW* age-standardized incidence rate by 1985 Segi’s world standard population, *ASMRC* age-standardized mortality rate by 2000 Chinese standard population, *ASMRW* age-standardized mortality rate by 1985 Segi’s world standard population

### Mortality estimation

About 21,320 NPC death cases, accounting for 0.96% of all cancer death cases in 2013 in China, were estimated to occur in China that year; its crude mortality, ASMRC, and ASMRW were 1.57/100,000, 1.10/100,000, and 1.08/100,000, respectively. About 15,730 male NPC death cases, accounting for 1.12% of all male cancer death cases in 2013 in China, were estimated to occur in China that year. The crude mortality, ASMRC, and ASMRW were 2.26/100,000, 1.63/100,000, and 1.60/100,000, respectively. About 5580 female NPC death cases, accounting for 0.68% of all female cancer death cases in 2013 in China, were estimated to occur in China that year. The crude mortality, ASMRC, and ASMRW were 0.84/100,000, 0.58/100,000, and 0.56/100,000, respectively. The male mortality was much higher than the female mortality (Table 5). The ASMRW in urban, rural, and both areas in males were 3.12, 2.59, and 2.86 times of those in females, respectively. The mortality in the Western area was the highest, followed by those in the Middle and Eastern areas, with the mortality in the Middle area slightly higher than that in the Eastern area.

**Table 5:** NPC mortality in China in 2013

Areas	Sex	CR (1/10^5^)	Prop (%)	ASMRC (1/10^5^)	ASMRW (1/10^5^)	Cum rate (%)	TASR (1/10^5^)	Rank
All	Both	1.57	0.96	1.10	1.08	0.12	2.09	18
	Male	2.26	1.12	1.63	1.60	0.19	3.10	13
	Female	0.84	0.68	0.58	0.56	0.06	1.06	18
Urban areas	Both	1.61	1.00	1.09	1.07	0.12	2.10	19
	Male	2.38	1.21	1.65	1.62	0.19	3.16	14
	Female	0.81	0.65	0.54	0.52	0.06	1.01	19
Rural areas	Both	1.51	0.91	1.12	1.09	0.13	2.08	18
	Male	2.12	1.02	1.62	1.58	0.18	3.02	12
	Female	0.87	0.71	0.63	0.61	0.07	2.13	16
Eastern areas	Both	1.62	0.86	1.02	1.00	0.12	3.27	17
	Male	2.35	1.01	1.51	1.49	0.18	0.95	13
	Female	0.87	0.61	0.53	0.52	0.06	2.02	18
Middle areas	Both	1.41	0.91	1.06	1.03	0.11	2.98	19
	Male	2.05	1.06	1.59	1.55	0.17	1.03	13
	Female	0.75	0.64	0.54	0.53	0.06	3.21	18
Western areas	Both	2.15	1.38	1.63	1.59	0.18	4.82	15
	Male	3.07	1.54	2.36	2.29	0.27	1.55	10
	Female	1.20	1.07	0.91	0.89	0.10	2.13	15

*CR* crude rate, *Prop* proportion, *ASMRC* age-standardized mortality rate by Chinese standard population, *ASMRW*, age-standardized mortality rate by 1985 Segi’s world standard population, *Cum rate* cumulative rate for patients aged 0–74 years, *TASR* truncated age-standardized rate for patients aged 35–64 years, *Rank* rank of NPC mortality in all cancers’ mortalities

### Age-specific mortality

**Fig. 2:**
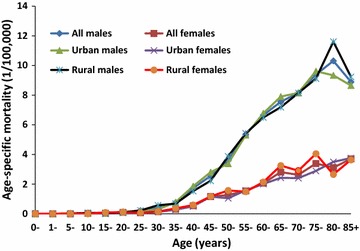
NPC age-specific mortality in China in 2013. The age-specific mortality rises quickly from age 35–39 years, peaks at age 80–84 for males and 85+ years for females. The mortalities in males are much higher than those in females. The patterns of male and female age-specific mortalities in urban and rural areas are basically the same

NPC age-specific mortalities in China in 2013 began to rise quickly from age 35–39 years, peaked at age 80–84 years for males and at age 85+ years for females, and down thereafter. The male mortality was obviously higher than the female mortality. The trends of male and female age-specific mortalities in urban and rural areas were basically the same, with only slight difference, such as the urban male and rural female mortalities peaked at age 75–79 years (Fig. 2). The age-specific mortalities of NPC varied at some degree in different Chinese areas, e.g., it peaked at age 65–69 years for males in the Western area and at 75–79 years for females in the Middle area.

### Mortality differences between regions

About 11,780 NPC death cases, accounting for 1.00% of all cancer death cases in Chinese urban areas in 2013, were estimated to occur in Chinese urban areas that year. The crude mortality, ASMRC, and ASMRW were 1.61/100,000, 1.09/100,000, and 1.07/100,000, respectively. About 9540 NPC death cases, accounting for 0.91% of all cancer-related death cases in Chinese rural areas in 2013, were estimated to occur in Chinese rural areas that year. The crude mortality, ASMRC, and ASMRW were 1.51/100,000, 1.12/100,000, and 1.09/100,000, respectively (Table 3). The mortalities in urban areas were close to those in rural areas, with the crude mortality slightly higher but the ASMRC and ASMRW slightly lower in urban areas (Table 5).

Generally, the highest mortality was seen in the Western area, followed by those in the Middle and Eastern areas in China in 2013, but with no significant difference; the same trend was found in rural areas. However, in urban areas, the highest rates were seen in the Western area, followed by the Eastern and Middle areas, although without significant difference too.

Among the Chinese seven administrative regions, the mortality in South China was the highest, followed by those in Southwest, Central, East, Northwest, Northeast, and North China. Notably, the ASMRW (4.92/100,000) in South China was 3.8 times higher than that in Southwest China (1.31/100,000), in which the mortality was the second highest.

In terms of ASMRW, top 3 death provinces in the 31 provinces, autonomous regions, and municipalities directly under the central government in China in 2013 were Guangxi, Guangdong, and Hainan, in which the ASMRWs were 5.01/100,000, 4.99/100,000, and 3.27/100,000, respectively, all located in South China. Guizhou, Sichuan, Chongqing, Shanghai, and Hubei also ranked the top 10 mortality provinces, which do not locate in South China (Table 3). Top 3 mortality areas in the 255 domestic cancer registries in 2013 were Cangwu county of Guangxi autonomous region, Sihui city of Guangdong province, and Longnan county of Jiangxi province, in which the ASMRWs were 14.24/100,000, 12.54/100,000, and 11.16/100,000, respectively. Top 10 mortality areas all located in South China.

## Discussions

Compared with the data of GLOBOCAN 2012 [2], NPC incidence and mortality in China in 2013 (ASIRW and ASMRW: 2.31/100,000 and 1.10/100,000, respectively) were lower than those in Southeast Asia (4.3/100,000 and 2.5/100,000, respectively), significantly higher than those in the world (1.2/100,000 and 0.7/100,000, respectively), higher than those in East Asia (1.8/100,000 and 1.0/100,000, respectively); the incidence higher but mortality lower than those in China in 2012 (1.9/100,000 and 1.2/100,000, respectively). According to GLOBOCAN 2012, NPC incidence and mortality in China in 2013 ranked the 16th and 32th among 184 countries and regions in 2012, respectively; the incidence was similar to those in Thailand (2.1/100,000) and Bhutan (2.2/100,000), and the mortality was similar to those in Guam (1.1/100,000) and Timor-Leste (1.1/100,000). It indicated that NPC incidence and mortality in China in 2013 were still at high levels worldwide, although they both were lower than those in China in 2010 [1].

Compared with the study we carried out in 2014 [1], NPC incidence in China was lower in 2013 than in 2010, the crude incidence, ASIRC, and ASIRW decreased by 2.21, 11.15, and 11.07%, respectively. The same results for the incidence were found in urban and rural areas. NPC crude mortality in China was slightly higher in 2013 than in 2010 (increased by 2.61%), but the ASMRC and ASMRW were slightly lower in 2013 than in 2010 (decreased by 8.33% and 8.47%, respectively). The same trends of mortality were found for rural areas. In urban areas, the rates were all lower in 2013 than in 2010. The incidences and mortalities were all not significantly different between the above 2 years. It revealed that comparing with the rates in 2010, NPC incidence and mortality in China in 2013 witnessed no obvious decreasing or increasing trend. This was not consistent with the results of the research by Xu et al. [13], which showed a decreasing mortality trend in China.

One of the epidemiological characteristics of NPC is that its incidence and mortality varied by geography, sex, and age. However, the present study revealed that there was no substantial difference in NPC incidence and mortality between Chinese urban and rural areas, which was basically consistent with the results in our previous study [1], suggesting that there were also no marked differences in factors linked to NPC incidence and mortality in urban and rural areas. Our study also showed that NPC incidence and mortality were the highest in the Western area, followed by the Middle and Eastern areas, but without significant differences, which was basically consistent with the results of previous results [1] and contrast to the results of the third national all-death-causes survey in China [5], and the reasons might be the same as those referred in our previous study [1], i.e., the distribution of sampling places may greatly influence the results. NPC geographic distribution features could be better described through stratification into seven administrative regions [1]. In the present study, NPC incidence and mortality were also significantly higher in South China than in other Chinese regions; the rates in South China were 3.4 and 3.8 times higher than those in the region with the second highest rates, respectively, and these findings were consistent with our previous reports too [1].

Our present study also showed that top 3 incidence provinces in 31 provinces, autonomous regions, and municipalities directly under the central government of China in 2013 were Guangxi, Guangdong, and Hunan, respectively, and top 3 mortality provinces were Guangxi, Guangdong, and Hainan, respectively—all locate in South China. It should be noticed that some provinces such as Tibet, Sichuan, Chongqing, Shanghai, and Hubei, which do not locate in South China, also ranked top 10 incidence and mortality provinces. Top 10 incidence and mortality areas among the 255 cancer registry areas in 2013 all locate in South China. Cangwu county of Guangxi autonomous region, Sihui city of Guangdong province, Longnan county of Jingxi province, and Mayang county of Hunan province were among the top 3 incidence or mortality areas. Zhongshan city of Guangdong province, which ranked the 1st in CIF 9 and 10 [23, 24], only ranked the 4th among the 255 cancer registry areas. However, Nanxiong city of Guangdong province and Longnan county of Jiangxi province, which are adjacent to each other geographically, also had high NPC incidence and mortality.

NPC incidence and mortality varied remarkably by sex and age. We determined that NPC incidence and mortality were significantly higher in males than in females, which was consistent with the results of previous reports [1–18]. Some differences existed between the age-specific incidences in the present study and our previous studies [1]. The female age-specific incidence peaked at age 80–84 in the present study but at age 60–64 in our previous study [1]. Difference in age-specific mortality between the present study and our previous study [1] was more obvious. The male and female age-specific mortalities were not close to each other at older age groups. The male age-specific mortalities increased quickly from age 30–34, remained stable after age 60, and peaked at age 80–84 in our previous study [1], but increased quickly from 35–39 and peaked at 80–84 in the present study. The female age-specific mortalities increased more quickly after age 75–79 and peaked at age 85+ in our previous study [1], but peaked at 75–79 and remained stable thereafter in the present study.

Although the data quality of NPC in this study were better than that in our previous study in 2014 [1], that in the 2012 Chinese Cancer Registry Annual Report [25, 26], and that of all cancers in 2013 in China [19, 20], the MV% of NPC data in this study was still lower than that of NPC data in 2003–2007 Cancer Incidence and Mortality in China (79.14%) [27], much lower than that of NPC data in CIF 9 (male, 96.28%) [23] and CIF 10 (female, 83%) [24], and also much lower than that of Zhongshan [17, 18]. Moreover, NPC should have a high MV%, as the nasopharynx is easy to be biopsied. All this indicated that NPC data quality in this study remained to be improved.

In conclusion, although NPC data quality in this study remained to be improved, the distribution or location of the selected population-based cancer registries could influence the results of the study, and the current results showed that NPC incidence and mortality in China in 2013 were still at high level worldwide, and these were consistent with the results of previous reports. NPC incidence and mortality in this study were close to the rates of our previous study in 2014, indicating that NPC incidence and mortality witnessed no obvious increasing or decreasing trend. Obvious differences of geography, sex, and age existed for the NPC incidence and mortality in this study, consistent with previous reports too. This study is very useful and helpful for the control and prevention of NPC in China, and enlightens the researches on NPC in the future.
